# Return to competition after ACL reconstruction: Factors influencing rates and timing in Swedish football players

**DOI:** 10.1002/ksa.12579

**Published:** 2025-01-26

**Authors:** Alexander Sandon, Joanna Kvist, Henrik Hedevik, Magnus Forssblad

**Affiliations:** ^1^ Department of Molecular Medicine & Surgery, Stockholm Sports Trauma Research Center Karolinska Institute Stockholm Sweden; ^2^ Division of Physiotherapy, Department of Health, Medicine and Caring Science Linköping University Linköping Sweden

**Keywords:** ACL, ACL reconstruction, football, return to competition, return to sports, soccer

## Abstract

**Purpose:**

To investigate the rate and timing for return to football league games after anterior cruciate ligament reconstruction (ACLR) in Swedish players, examining associations with sex, age, level, graft and additional ACL surgery.

**Method:**

Data from the Swedish National Knee Registry (SNKLR) and the Swedish Football Association's IT System (FOGIS) were used. The study cohort comprised 971 football players, 64% males, who underwent primary ACLR. Demographics, graft type and surgical information were extracted from the SNKLR and game participation from FOGIS. Follow‐up for return to competition (RTC) was conducted for 36 months, while additional ACLR follow‐up was 3–7 years. Statistical analyses, including Kaplan–Meier survival curves and relative risk calculations, were employed to assess factors influencing RTC rates and timing.

**Results:**

Out of 971 players analyzed, 53% RTC within 3 years with no difference between males and females, at a mean of 15 months (median 14 months) from surgery to the first game. Eleven (2%) players RTC < 6 months from ACLR, 62 (12%) 6–9 months, 125 (24%) 9–12 months and 331 (63%) >12 months. Patellar tendon (PT) grafts demonstrated superior performance, showing quicker returns and higher RTC rates (*p* = 0.005) compared to hamstring (hazard ratio [HR]: 0.63 [0.48–0.84]) and quadriceps tendon grafts (HR: 0.53 [0.30–0.93]). Players competing in higher divisions pre‐injury experienced significantly swifter and higher RTC rates (*p* < 0.001). Ninety‐five (10%) had a registered additional ACLR. Players who RTC did not exhibit a significantly higher rate of revision (35 [7%] vs. 25 [5%]). However, those who returned faced a heightened risk of contralateral ACLRs compared to those who did not RTC (32 [6%] vs. 4 [1%] RR 1.72 [1.59–1.96], *p* < 0.001).

**Conclusion:**

The study reveals that 53% of football players RTC after ACLR, predominantly after more than 12 months. The RTC was higher and faster in high‐level players and those receiving a PT graft. The slow RTC may contribute to the relatively low rate of additional ACLRs.

**Level of Evidence:**

Level III.

**Abbreviations**
ACL: anterior cruciate ligament
ACLR: anterior cruciate ligament reconstruction
CACLR: contralateral anterior cruciate ligament reconstruction
CI: confidence interval
FOGIS: Swedish Football Association's IT System
HR: hazard ratio
HT: hamstring tendon
IQR: interquartile range
PT: patellar tendon
QT: quadriceps tendon
RR: relative risk
RTC: return to competition
RTS: return to sports
SD: standard deviation
SNKLR: Swedish National Knee Ligament Registry

## INTRODUCTION

In Sweden, 37% of all surgically treated anterior cruciate ligament (ACL) injuries occur during football and the injury results in a long time of absence from play, affecting both the player [19] and the team [24]. The majority of athletes expect to return to sport (RTS) after the injury [14, 44, 49], but not all manage to RTS rates have been described to be 55%–83% [3, 33], with higher rates for elite athletes [33]. The rates for return to play are influenced by age, where younger football players are more likely to RTS [30, 40], and sex, where female athletes have lower odds to RTS compared to males [10]. In football, RTS rates are also influenced by the level of play, with a variation between 21% [37] and 97%, with elite football players reporting an 80%–97% RTS [1, 8, 20, 28, 47]. However, football players who have RTS have a decreased performance level, at least during the first seasons after ACL reconstruction (ACLR) [1, 20, 45]. The definition of RTS differs in many studies from self‐reported RTS [3] to registered participation in games (return to competition, RTC) [47] at the same level of performance as before the injury [20]. Another important factor in the RTS is the high rate of additional ACLR seen in football players [15, 16, 40].

The numbers and timing of RTC after ACLR for football players at different levels of participation are yet unknown. Understanding the factors that affect return to football competition rates and the time to RTC after ACLR, including graft choice, can inform clinical decision‐making, guide rehabilitation protocols and give the players and the coaching staff realistic expectations on the RTC. Thus, this study aimed to investigate numbers and timing for return to football league games after ACLR and their associations to sex, age, level of competition, graft and additional ACL surgery. The hypothesis is that football players who undergo ACLR are more likely to return to competitive league games within a shorter time frame if they are younger, male and playing at an elite level with graft choice potentially influencing the timing of their return.

## METHODS

Patient selection for this study involved identifying individuals within the Swedish National Knee Registry (SNKLR) who had a questionnaire in the registry directed to ACL‐injured football players, from which descriptive data have been disseminated in previous publications [41]. Inclusion criteria necessitated a documented unilateral primary ACLR in the SNKLR, along with active registration within a Swedish football league team and possessing a valid license within the Swedish Football Association's IT System (FOGIS). Exclusion criteria involved patients with other knee ligament injuries surgically treated, allografts, individuals younger than 15 at the time of surgery (as per ethical approval) and those with nerve injuries. The study received approval from the Swedish Ethical Review Authority (ID 2024‐00159‐02).

### Data collection

To gather relevant information, registry data were extracted from both the SNKLR and FOGIS. The participants' social security number was utilized to enable seamless data extraction and facilitate comparisons between the two registries. Inclusivity extended to both male and female football players who met the outlined study criteria. The study has a follow‐up of 36 months from the ACLR in terms of RTC and between 3 and 7 years from the ACLR in terms of additional ACLRs.

### Swedish National Knee Ligament Registry

Established in 2005 as a surgical registry for ACL injuries, the SNKLR contains orthopaedic surgeon‐entered surgical data [2, 31]. It encompasses records for approximately 90% of all ACL surgeries conducted in Sweden. Additionally, the SNKLR incorporates self‐reported data and patient‐reported outcome measures related to knee function and quality of life. These measures are recorded both pre‐surgery and at 1, 2, 5 and 10 years post‐ACLR.

### Swedish Football Association's IT System

The administrative IT system of the Swedish Football Association, FOGIS, serves as a comprehensive repository of information regarding player participation in Swedish football league games. To participate in league games, all players must be registered with a football association, and those aged 15 or older must possess a registered license in FOGIS. Data extracted from FOGIS included the date of all games the players have participated in, providing essential insights into the timeline of RTC after the procedure.

### Statistics

Statistical analyses were performed using IBM SPSS Statistics for Windows (Version 29.0.2.0 Armonk, NY: IBM Corp) to investigate various aspects of RTC after ACLR in football players. Relative risk (RR) with 95% confidence interval (CI) calculations were employed to compare RTC rates among different variables and to assess the incidence of additional ACLR between players who RTC and those who did not. Descriptive statistics, including means, medians and percentages, were utilized to summarize key findings such as the proportion of players returning to competition within specific post‐surgery time frames and the distribution of graft types among the study population. Age at ACLR was categorized into six groups: 15–20, 21–25, 26–30, 31–35 and 36 years and older. Statistical significance was determined at a *p* value <0.05. Sex‐specific associations were investigated through separate analyses for female and male players. Survival analyses were performed using R Statistical Software (v4.3.2; R Core Team 2021) to create Kaplan–Meier curves on RTC to assess the time to return to football among those with different graft types or playing at different levels of competition pre‐injury. Hazard ratio (HR) with 95% CI was calculated using Cox proportional hazards regression models. The proportional hazards assumption was checked using Schoenfeld residuals.

## RESULTS

The results emanate from an analysis of 971 football players who underwent ACLR, comprising 623 (64%) males and 348 (36%) females. RTC and time to RTC for the study variables can be found in Table 1. Fifty‐three per cent of the players successfully RTC post‐ACLR within 3 years, with a mean time lapse of 15 (SD 6) months from surgery to the first game (Table 2). Eleven (2%) players RTC within 6 months from the ACLR, 62 (12%) returned within 6–9 months, 125 (24%) returned between 9 and 12 months and 331 (63%) RTC after more than 12 months. There were similar rates of RTC for male and female football players (52% vs. 55%). The males were on average 3 years older at the time of the ACLR than the females, 24 versus 21, respectively.

**Table 1:** Return to competition (RTC) within 36 months after ACLR.

					Months (mean and interval) from ACLR to RTC				
		Total	RTC	RR (95% CI)		3–6	6–9	9–12	>12
Players	Factors	*N* (col %)	*N* (%)	Overall *p*‐value	Mean ± SD	*N* (%)	*N* (%)	*N* (%)	*N* (%)
All players		971 (100%)	515 (53%)						
	Sex			n.s.					
	Male	623 (64%)	325 (52%)	1	15 ± 6	8 (2%)	37 (11%)	84 (26%)	196 (60%)
	Female	348 (36%)	190 (55%)	1.05 (0.93–1.18)	15 ± 6	3 (2%)	25 (13%)	41 (22%)	121 (64%)
	Age interval at ACLR			*p* < 0.001					
	15–20 years (Ref)	397 (41%)	237 (60%)	1	15 ± 6	1 (0%)	21 (9%)	58 (24%)	157 (66%)
	21–25 years	262 (27%)	146 (56%)	0.93 (0.82–1.07)	15 ± 7	7 (5%)	21 (14%)	39 (27%)	79 (54%)
	26–30 years	188 (19%)	86 (46%)	0.77 (0.64–0.91)	15 ± 7	1 (1%)	15 (17%)	17 (20%)	53 (62%)
	31–35 years	75 (8%)	34 (45%)	0.76 (0.58–0.99)	16 ± 6	2 (6%)	2 (6%)	8 (24%)	22 (65%)
	36+ years	49 (5%)	12 (24%)	0.41 (0.25–0.68)	14 ± 8	0 (–)	3 (25%)	3 (25%)	6 (50%)
	Level of play			*p* < 0.001					
	Top (Ref)^a^	59 (6%)	49 (83%)	1	13 ± 5	0 (–)	4 (8%)	20 (41%)	25 (51%)
	High (Ref)^a^	205 (21%)	132 (64%)	1	15 ± 6	3 (2%)	15 (11%)	31 (23%)	83 (63%)
	Middle	253 (26%)	148 (58%)	0.85 (0.75–0.97)	15 ± 6	0 (–)	18 (12%)	38 (26%)	92 (63%)
	Low	353 (36%)	172 (49%)	0.71 (0.62–0.81)	15 ± 7	8 (5%)	25 (15%)	35 (20%)	104 (60%)
	Recreational	100 (10%)	14 (14%)	0.20 (0.12–0.33)	20 ± 6	0 (–)	0 (–)	1 (7%)	13 (93%)
	ACLR graft			*p* = 0.009					
	Patellar tendon (Ref)	79 (8%)	53 (67%)	1	13 ± 4	1 (2%)	9 (17%)	12 (23%)	31 (58%)
	Hamstring tendon	858 (88%)	446 (52%)	0.77 (0.66–0.92)	15 ± 6	10 (2%)	52 (12%)	110 (25%)	271 (61%)
	Quadriceps tendon	34 (4%)	16 (47%)	0.70 (0.48–1.03)	18 ± 8	0 (–)	1 (6%)	3 (19%)	12 (75%)
Male players	Age interval at ACLR			*p* = 0.003					
	15–20 years (Ref)	196 (31%)	118 (60%)	1	15 ± 6	1 (1%)	10 (8%)	31 (26%)	76 (64%)
	21–25 years	188 (30%)	106 (56%)	0.94 (0.79–1.11)	15 ± 7	5 (5%)	13 (12%)	32 (30%)	56 (53%)
	26–30 years	141 (23%)	64 (45%)	0.75 (0.61–0.93)	15 ± 6	1 (2%)	9 (14%)	13 (20%)	41 (64%)
	31–35 years	60 (10%)	27 (45%)	0.75 (0.55–1.01)	16 ± 6	1 (4%)	2 (7%)	6 (22%)	18 (67%)
	36+ years	38 (6%)	10 (26%)	0.44 (0.25–0.75)	13 ± 6	0 (–)	3 (30%)	2 (20%)	5 (50%)
	Level of play			*p* < 0.001					
	Top (Ref)^a^	31 (5%)	26 (84%)	1	13 ± 5	0 (–)	1 (4%)	10 (38%)	15 (58%)
	High (Ref)^a^	64 (10%)	40 (63%)	1	14 ± 6	1 (3%)	5 (13%)	13 (33%)	21 (53%)
	Middle	186 (30%)	110 (59%)	0.85 (0.71–1.02)	15 ± 6	0 (–)	12 (11%)	32 (29%)	66 (60%)
	Low	260 (42%)	139 (53%)	0.77 (0.65–0.92)	16 ± 7	7 (5%)	19 (14%)	28 (20%)	85 (61%)
	Recreational	81 (13%)	10 (12%)	0.18 (0.10–0.32)	20 ± 7	0 (–)	0 (–)	1 (10%)	9 (90%)
	ACLR graft			*p* = 0.011					
	Patellar tendon (Ref)	43 (7%)	30 (70%)	1	12 ± 4	0 (–)	6 (20%)	10 (33%)	14 (47%)
	Hamstring tendon	554 (89%)	284 (51%)	0.73 (0.59–0.91)	15 ± 7	8 (3%)	30 (11%)	72 (25%)	174 (61%)
	Quadriceps tendon	26 (4%)	11 (42%)	0.61 (0.37–0.99)	17 ± 8	0 (–)	1 (9%)	2 (18%)	8 (73%)
Female players	Age interval at ACLR			n.s.					
	15–20 years (Ref)	201 (58%)	119 (59%)	1	16 ± 6	0 (–)	11 (9%)	27 (23%)	81 (68%)
	21–25 years	74 (21%)	40 (54%)	0.91 (0.72–1.16)	14 ± 6	2 (5%)	8 (20%)	7 (18%)	23 (58%)
	26–30 years	47 (14%)	22 (47%)	0.79 (0.57–1.10)	15 ± 8	0 (–)	6 (27%)	4 (18%)	12 (55%)
	31–35 years	15 (4%)	7 (47%)	0.79 (0.45–1.37)	14 ± 5	1 (14%)	0 (–)	2 (29%)	4 (57%)
	36+ years	11 (3%)	2 (18%)	0.31 (0.09–1.08)	23 ± 16	0 (–)	0 (–)	1 (50%)	1 (50%)
	Level of play			*p* < 0.001					
	Top (Ref)^a^	28 (8%)	23 (82%)	1	13 ± 4	0 (–)	3 (13%)	10 (43%)	10 (43%)
	High (Ref)^a^	141 (41%)	92 (65%)	1	16 ± 6	2 (2%)	10 (11%)	18 (20%)	62 (67%)
	Middle	67 (19%)	38 (57%)	0.83 (0.66–1.05)	16 ± 7	0 (–)	6 (16%)	6 (16%)	26 (68%)
	Low	93 (27%)	33 (35%)	0.52 (0.39–0.70)	14 ± 6	1 (3%)	6 (18%)	7 (21%)	19 (58%)
	Recreational	19 (5%)	4 (21%)	0.31 (0.13–0.74)	21 ± 5	0 (–)	0 (–)	0 (–)	4 (100%)
	ACLR graft			n.s.					
	Patellar tendon (Ref)	36 (10%)	23 (64%)	1	15 ± 5	1 (4%)	3 (13%)	2 (9%)	17 (74%)
	Hamstring tendon	304 (87%)	162 (53%)	0.83 (0.64–1.09)	15 ± 6	2 (1%)	22 (14%)	38 (23%)	100 (62%)
	Quadriceps tendon	8 (2%)	5 (63%)	0.98 (0.54–1.77)	20 ± 8	0 (–)	0 (–)	1 (20%)	4 (80%)

Abbreviations: ACLR, anterior cruciate ligament reconstruction; CI, confidence interval; n.s, not significant; Ref, reference category; RR, risk ratio.

^a^ Top and high level of play is combined when analyzing.

**Table 2:** Characteristics of male and female football players related to ACLR graft.

		Male football players					Female football players				
	All players	Total	PT	HT	QT	*p*‐value	Total	PT	HT	QT	*p*‐value
RTC, *n* (%)	515 (53%)	325 (52%)	30 (70%)	284 (51%)	11 (42%)	*p* = 0.038	190 (55%)	23 (64%)	162 (53%)	5 (63%)	n.s.
Months to RTC											
Mean ± SD	15 ± 6	15 ± 6	12 ± 4	15 ± 7	17 ± 8	*p* = 0.029	15 ± 6	15 ± 5	15 ± 6	20 ± 8	n.s.
Median (IQR)	14 (11–18)	14 (11–18)	12 (10–15)	14 (11–19)	15 (10–18)	*p* = 0.050	14 (11–19)	16 (11–18)	14 (11–19)	18 (16–19)	n.s.
Interval, *n* (%)						n.s.					n.s.
3–6 months	11 (2%)	8 (2%)	0 (–)	8 (3%)	0 (–)		3 (2%)	1 (4%)	2 (1%)	0 (–)	
6–9 months	62 (12%)	37 (11%)	6 (20%)	30 (11%)	1 (9%)		25 (13%)	3 (13%)	22 (14%)	0 (–)	
9–12 months	125 (24%)	84 (26%)	10 (33%)	72 (25%)	2 (18%)		41 (22%)	2 (9%)	38 (23%)	1 (20%)	
>12 months	317 (62%)	196 (60%)	14 (47%)	174 (61%)	8 (73%)		121 (64%)	17 (74%)	100 (62%)	4 (80%)	
Age at ACLR, mean ± SD	23 ± 6	24 ± 6	24 ± 6	24 ± 6	21 ± 5	*p* = 0.026	21 ± 6	22 ± 6	21 ± 6	19 ± 3	n.s.
Age interval at ACLR, *n* (%)						n.s.					n.s.
15–20 years	397 (41%)	196 (31%)	13 (30%)	168 (30%)	15 (58%)		201 (58%)	23 (64%)	173 (57%)	5 (63%)	
21–25 years	262 (27%)	188 (30%)	14 (33%)	167 (30%)	7 (27%)		74 (21%)	6 (17%)	65 (21%)	3 (38%)	
26–30 years	188 (19%)	141 (23%)	8 (19%)	130 (23%)	3 (12%)		47 (14%)	4 (11%)	43 (14%)	0 (–)	
31–35 years	75 (8%)	60 (10%)	6 (14%)	54 (10%)	0 (–)		15 (4%)	1 (3%)	14 (5%)	0 (–)	
36+ years	49 (5%)	38 (6%)	2 (5%)	35 (6%)	1 (4%)		11 (3%)	2 (6%)	9 (3%)	0 (–)	
Level of play, *n* (%)						*p* < 0.001					n.s.
Top	59 (6%)	31 (5%)	8 (19%)	19 (3%)	4 (16%)		28 (8%)	5 (14%)	22 (7%)	1 (13%)	
High	205 (21%)	64 (10%)	4 (9%)	54 (10%)	6 (24%)		141 (41%)	13 (36%)	123 (40%)	5 (63%)	
Middle	253 (26%)	186 (30%)	14 (33%)	168 (30%)	4 (16%)		67 (19%)	9 (25%)	57 (19%)	1 (13%)	
Low	353 (36%)	260 (42%)	15 (35%)	241 (44%)	4 (16%)		93 (27%)	6 (17%)	87 (29%)	0 (–)	
Recreational	100 (10%)	81 (13%)	2 (5%)	72 (13%)	7 (28%)		19 (5%)	3 (8%)	15 (5%)	1 (13%)	
Additional ACLR, *n* (%)	95 (10%)	52 (8%)	3 (7%)	45 (8%)	4 (15%)	n.s.	43 (12%)	4 (11%)	39 (13%)	0 (–)	n.s.
Revision	60 (6%)	36 (6%)	2 (5%)	31 (6%)	3 (12%)	n.s.	24 (7%)	0 (–)	24 (8%)	0 (–)	n.s.
CACLR	36 (4%)	17 (3%)	1 (2%)	15 (3%)	1 (4%)	n.s.	19 (5%)	4 (11%)	15 (5%)	0 (–)	n.s.

Abbreviations: ACLR, anterior cruciate ligament reconstruction; CACLR, contralateral anterior cruciate ligament reconstruction; HT, hamstring tendon; IQR, Interquartile range; n.s., not significant; PT, patellar tendon; QT, quadriceps tendon; RTC, return to competition; SD, standard deviation.

**Figure 1:**
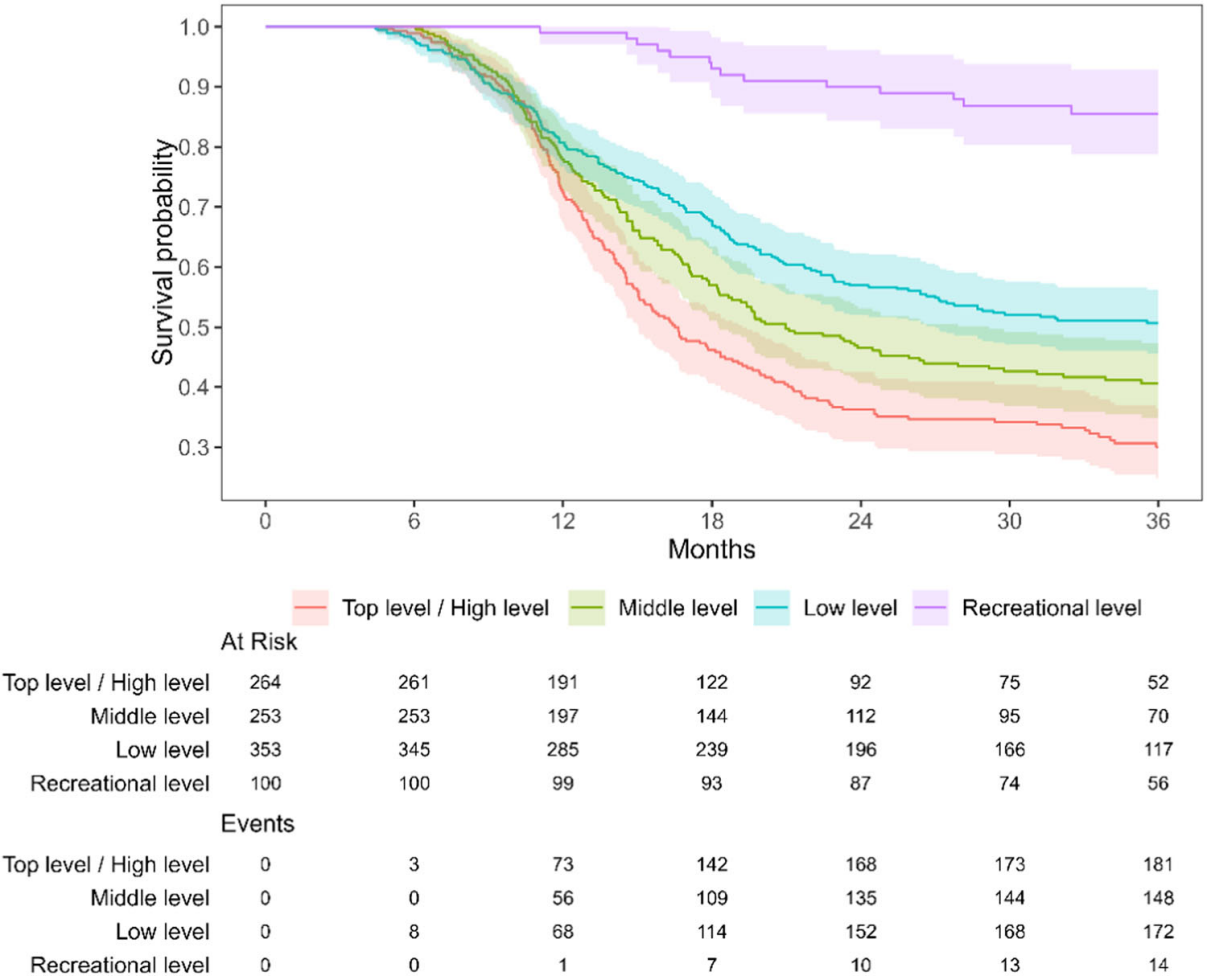
Kaplan–Meier graph of the rate and time to RTC after ACLR for football players at different levels. Months represent the time from surgery to RTC. At Risk represents players who have not yet RTC and Events is players who have RTC. ACLR, anterior cruciate ligament reconstruction; RTC, return to competition.

Players who competed in top divisions before their ACLR had a higher RTC rate compared to their counterparts from lower divisions (83% vs. 14%–64%, *p* < 0.001). The higher RTC depending on the preinjury level of play was seen in both male (*p* < 0.001) and female (*p* < 0.001) players. Figure 1 illustrates the faster and higher RTC for higher‐level players demonstrated by the Kaplan–Meier analysis.

When considering age, players in the youngest age groups (15–20 years) exhibited a significantly (*p* < 0.001) higher RTC rate for the male players but there was no significant difference in the RTC rates for the different age groups for the females. However, in the female players, only 18% of those aged 36+ at the time of the surgery RTC within 36 months compared to 59% for those in the age group 15–20.

### Graft

**Figure 2:**
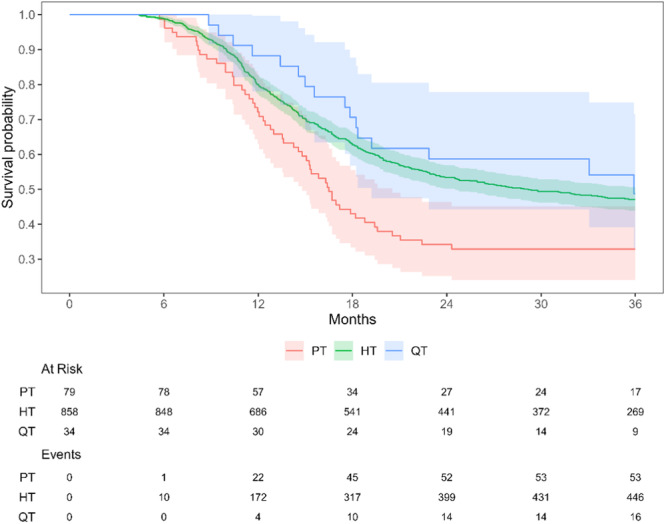
Kaplan–Meier graph of the rate and time to RTC after ACLR for football players for patellar tendon (PT), hamstring tendon (HT) and quadriceps tendon (QT) grafts. Months represent the time from surgery to RTC. At Risk represents players who have not yet RTC and Events is players who have RTC. ACLR, anterior cruciate ligament reconstruction; RTC, return to competition.

The dominant choice of graft was the hamstring tendon (HT), accounting for 88% of cases, followed by patellar tendon (PT) grafts at 8% and quadriceps tendon (QT) grafts at 4% (Table 1). A Kaplan–Meier analysis revealed that the PT grafts demonstrated both quicker and higher RTC rates (*p* = 0.005) compared to HT (HR: 0.63 [0.48–0.84]) and QT grafts (HR: 0.53 [0.30–0.93]) (Figure 2). For male players, the RTC rate was higher for PT grafts, 70% compared to HT 51% and QT grafts 42%. The average time from ACLR to the RTC differed across graft types: 12 months for PT, 15 months for HT and 17 months for QT grafts. The incidence of revisions among male players who received a QT was 12%, compared to 2% in the PT group and 6% in those with an HT graft after RTC; however, this difference did not reach statistical significance. For the female players, the average time from ACLR to the first football game was 15 months for both the PT and HT grafts and 20 months for players receiving a QT graft, with no significant difference between the groups. The relationship between different grafts and RTC is presented in Table 2.

### Additional ACLR

At a follow‐up period ranging from 3 to 7 years after the initial ACLR, 95 (10%) football players had a registered revision or contralateral ACLR (CACLR) recorded in the SNKLR. Specifically, 60 (6%) underwent revision ACLR, while 36 (4%) underwent CACLR (Table 2).

Players who RTC had an RR of 1.38 (1.19–1.59) for an additional ACLR. For males, the RR was 1.33 (1.08–1.63), while females exhibited a risk of 1.44 (1.17–1.77) (*p* = 0.007 and *p* < 0.001, respectively). No significant difference in revision rates was observed based on RTC status. However, for CACLR, a significantly heightened risk was identified among players who RTC, with an RR of 1.72 (1.59–1.96) (*p* < 0.001). Comparing RTC and non‐RTC groups, 14 (4%) male players experienced CACLR compared to three (1%) who did not (1.60 [1.27–2.03], *p* < 0.001). Similarly, among female players, the numbers were eighteen (9%) and one (1%), respectively (1.81 [1.56–2.10], *p* < 0.001). Additional ACLR incidences between RTC and non‐RTC groups are detailed in Table 3.

**Table 3:** Additional ACLR by RTC within 36 months.

			Additional ACLR		
			Yes	No	
Type of additional ACLR	Players	RTC within 36 months	*N* (row %)	*N* (row %)	RR (95% CI), *p*‐value
All ACLR	All	RTC	67 (13%)	448 (87%)	1.38 (1.19–1.59), *p* < 0.001
		No RTC	28 (6%)	428 (94%)	
	Male	RTC	35 (11%)	290 (89%)	1.33 (1.08–1.63), p = 0.007
		No RTC	17 (6%)	281 (94%)	
	Female	RTC	32 (17%)	158 (83%)	1.44 (1.17–1.77), *p* < 0.001
		No RTC	11 (7%)	147 (93%)	
Revision	All	RTC	35 (7%)	480 (93%)	1.11 (0.89–1.38), n.s.
		No RTC	25 (5%)	431 (95%)	
	Male	RTC	21 (6%)	304 (94%)	1.13 (0.85–1.50), n.s.
		No RTC	15 (5%)	283 (95%)	
	Female	RTC	14 (7%)	176 (93%)	1.07 (0.75–1.53), n.s.
		No RTC	10 (6%)	148 (94%)	
CACLR	All	RTC	32 (6%)	483 (94%)	1.72 (1.59–1.96), *p* < 0.001
		No RTC	4 (1%)	452 (99%)	
	Male	RTC	14 (4%)	311 (96%)	1.60 (1.27–2.03), p < 0.001
		No RTC	3 (1%)	295 (99%)	
	Female	RTC	18 (9%)	172 (91%)	1.81 (1.56–2.10), *p* < 0.001
		No RTC	1 (1%)	157 (99%)	

Abbreviations: ACLR, anterior cruciate ligament reconstruction; CACLR, contralateral anterior cruciate ligament reconstruction; CI, confidence interval; n.s., not significant; RR, risk ratio; RTC, return to competition.

## DISCUSSION

The most important finding of this study was that there were no sex differences in the timing or rates of return to competitive football (RTC) after ACLR among football players. Analyzing 971 cases, we found that 53% of players successfully resumed participation in official matches, a result consistent with previous studies on football players undergoing ACLR in Sweden [40, 43]. The study's design, which integrates surgical data with national football game participation records, provides a uniquely comprehensive follow‐up on RTC rates and timing across all levels of play.

We found no differences between male and female football players in rates or timing for RTC. Previous studies have shown high rates of RTP for both female National Football League [1] and male professional football [6, 9, 18, 45, 47], but to the best of our knowledge, studies comparing RTC rates between females and males in football at different competition levels are rare. Studies on RTSs in general have shown similar [51] or lower rates for RTS in females compared to males [3, 10, 30, 48]. In line with previous studies [30], we also found that there were age‐related sex differences. Klemm et al. [30] found significant differences in RTC between sexes in the 20‐ to 29‐year age group. In our study, males 15–20 years had higher RTC rates compared to older males. In the females, the youngest age group had a 59% RTC compared to 18% in the oldest age group, but this difference did not reach statistical significance. Many different factors, including knee function, functional performance and psychological and contextual factors, may affect the RTS [14, 43, 44, 46, 51].

Temporal aspects of RTC prominently unfold in our findings, revealing that 63% of players engaged in their first game after more than 12 months post‐surgery, with an additional 24% returning between 9 and 12 months. The mean RTC time stands at 15 months. Aligning with our results, Ardern et al. showcased a 33% RTC rate within 12 months among patients engaged in pivoting sports at a competitive level before ACLR [4]. The previous literature on football players has identified an RTC between 6 and 11 months, which is considerably faster; however, all of them were on professional players and on smaller cohorts [5, 6, 9, 18, 25, 27, 36, 47]. This discrepancy can be attributed to differences in study populations. Our study, encompassing players from all levels of play, distinctly illustrates that individuals competing in higher divisions before ACLR exhibit significantly swifter and higher return‐to‐play rates. This aligns with findings from a study on German players [45]. Younger players also show a faster and higher return. A higher RTC in younger [50] and higher‐level players has been well‐established [40]. Another possible contributing factor to the delayed RTC compared to previous studies could be a trend to allow the players to RTC later after the study by Grindem et al. [22] showing a reduced injury risk with RTC delayed for more than 9 months. Additionally, Kyritsis et al. [32] highlighted the significance of reaching rehabilitation milestones for a safer RTC, further influencing the timing of players' return.

Graft selection played a pivotal role, with PT grafts demonstrating superior performance. PT grafts exhibit a quicker return to football and a higher return rate compared to HT, which is in line with previous data [23, 38], and also compared to QT, which is a novel founding. A recent systematic review by DeFazio et al. [12] demonstrated a higher RTS for athletes receiving a PT over an HT graft while Bergeron et al. [7] found no difference in the return to baseline activity between the two grafts in their systematic review and meta‐analysis. There were also fewer revisions after the return to football for PT graft than in HT or QT graft although this difference did not reach statistical significance. Few revisions in athletes receiving a PT graft have been previously reported [29, 39]. QT autografts have become increasingly popular as primary grafts [11, 13, 35]. In this study, it exhibited a slower RTC and a lower return rate than the other grafts. This could possibly partly be explained by a slower rehabilitation of quadriceps strength [26]. While our study suggests that the PT autograft shows promising outcomes, caution is needed in interpreting these findings due to the overrepresentation of HT grafts in our cohort and the low number of QT grafts. Other factors, such as patient demographics, surgical techniques and rehabilitation protocols, may also contribute to observed outcomes and influence graft success.

The overall proportion of players experiencing additional ACLR in the form of a revision or CACLR remained relatively low at 10% in total and 13% for those that RTC compared to previous studies on ACL‐reconstructed football players. A 10‐year follow‐up on football players undergoing primary ACLR in 2005 and 2006 revealed additional ACL injuries in 22% of all players and 29% of those that RTC [40]. Among the female football cohort who returned to football, 42% had an additional ACL injury within 5–10 years, with two to five times higher risk compared to those who did not return to football play [17]. However, these studies also included those who did not undergo ACLR for their additional ACL injuries, which accounted for 29% in the study by Fältström et al. [17]. Similarly, a separate study focusing on 524 ACL‐reconstructed Swedish football talents highlighted that 23% of the players experienced a revision or CACLR [42]. These earlier findings underscored the substantial risks associated with resuming football activities post‐ACLR, emphasizing the need for comprehensive rehabilitation strategies and long‐term monitoring. However, our current study suggests a potential shift in outcomes, possibly attributable to advancements in surgical techniques, rehabilitation protocols and potentially delayed RTC. These factors may have collectively contributed to the observed decrease in the rate of additional ACLR among football players in our cohort. The observed risks associated with RTC are noteworthy; male players who RTC exhibited a 33% higher risk of undergoing additional ACLR, while females faced an even greater risk at 44%. The risk of CACLR for players who RTC was 60% higher for the male players and 81% higher for the female players. Surprisingly, no significant difference in revision rates was observed based on RTC status. Given the fact that football participation is a known risk activity for sustaining ACL injuries [8, 15, 21, 31], an increase in CACLR for those who RTC is expected, but it is unclear why the same was not observed regarding revisions. However, the same has been shown by Manara et al. [34] who found an increased risk of CACLR but not revisions for football players receiving an HT who RTS.

This study has several limitations that should be considered when interpreting the results. First, the retrospective cohort design introduces inherent biases, including potential selection bias and the inability to establish causality between variables. One notable limitation of this study is the uneven distribution of graft types used in ACLRs among the study cohort. The dominant choice of graft was HT, accounting for most cases, followed by PT and QT grafts. This uneven distribution may introduce bias in the interpretation of graft performance and outcomes. For example, the relatively low usage of certain grafts, such as QT grafts, compared to others may impact the generalizability of the findings and limit the ability to draw definitive conclusions about the performance of less commonly used grafts. Additionally, variations in surgical techniques associated with different graft types could influence outcomes, highlighting the need for further research to better understand the impact of graft selection on ACLR outcomes. Another notable limitation of this study is the potential underestimation of the true incidence of additional ACL injuries. The study relied on registered data for additional ACLRs, which may not capture all instances of additional surgery performed outside the national registry or those choosing not to have a new operation. Consequently, the reported incidence of additional ACL injuries, including revisions and CACLR, may be lower than the actual occurrence. This limitation could affect the accuracy of the findings regarding the long‐term outcomes of ACLRs and the overall risk of subsequent ACL injuries among football players. The SNKLR does not presently include any information about the rehabilitation undertaken nor is rehabilitation standardized across the country, which could have influenced RTC outcomes. Additionally, the seasonal nature of football in Sweden, with the lower divisions typically running from April to October, poses a significant limitation. This relatively short playing season may affect the timing of RTC after ACLR, particularly for players undergoing surgery during the off‐season. The limited duration of the playing season may influence rehabilitation timelines and return‐to‐play decisions, potentially impacting the observed RTC rates and timing. Therefore, the seasonal variability in football activity should be considered when interpreting the study findings, as it may introduce variability in RTC patterns and outcomes among players in different divisions and age groups.

## CONCLUSION

The study reveals that 53% of football players RTC after ACLR, predominantly after more than 12 months. Return rates were faster in high‐level players and those receiving a PT graft. The slower return rate may contribute to the relatively low rate of additional ACLRs.

## AUTHOR CONTRIBUTIONS

All authors contributed to the study conception and design. Material preparation, data collection and analysis were performed by all authors. The first draft of the manuscript was written by Alexander Sandon, and all authors commented on previous versions of the manuscript. All authors read and approved the final manuscript.

## CONFLICT OF INTEREST STATEMENT

The authors declare no conflicts of interest.

## ETHICS STATEMENT

The study is approved by the Swedish Ethical Review Authority, ID 2021‐07044‐01. All participants give their informed consent when entering the register.

## Data Availability

The full original surgical data can be extracted from the Swedish Knee Ligament Register (www.aclregister.nu) and football participation by the Swedish FA. The analyses involved in this manuscript can be provided by the first author.
